# Patient-specific numerical investigation of the correction of cervical kyphotic deformity based on a retrospective clinical case

**DOI:** 10.3389/fbioe.2022.950839

**Published:** 2022-09-09

**Authors:** Tianchi Wu, Hongyu Chen, Yu Sun, Tian Xia, Feifei Zhou, William W. Lu

**Affiliations:** ^1^ Department of Orthopaedics and Traumatology, The University of Hong Kong, Hong Kong, Hong Kong SAR, China; ^2^ Shenzhen Institutes of Advanced Technology, Chinese Academy of Science, Shenzhen, China; ^3^ Department of Orthopaedics, Peking University Third Hospital, Beijing, China; ^4^ Engineering Research Center of Bone and Joint Precision Medicine, Beijing, China; ^5^ Beijing Key Laboratory of Spinal Disease Research, Beijing, China

**Keywords:** patient-specific, numerical investigation, kyphosis correction, principal strain, range of motion

## Abstract

Little research has been reported on evaluating the safety of the fixation construct in cervical kyphosis correction. In this study, we proposed a principal-strain criterion to evaluate the safety of the fixation construct and validated the modeling method against a retrospective case of anterior cervical discectomy fusion (ACDF). From C2 to T2 vertebra bodies, fixation instruments were reconstructed and positioned as per postoperative computed tomography (CT) scans. Head weight (HW) and various moments estimated from isometric strength data were imposed onto the C2. The postoperative stability of non-surgical segments, deformations surrounding the screw trajectories, and contact slipping on zygapophysial joints were analyzed. The model was validated against the reality that the patient had a good fusion and deformity correction. The ACDF restricted the range of motions (ROMs) of cervical segments and lent stability to vertebra fusion, no failure was found in the finite element (FE) model of cervical vertebrae. The deformation surrounding the screw trajectories were concentrated to the lateral sides of trajectories, recommending that the shape of the anterior cervical plate conforming to the curvature of the vertebra and screws fully inserted into vertebrae reduced the deformation concentration around the screw trajectories.

## 1 Introduction

Cervical kyphotic deformity alters the normal functioning of the cervical spine, reducing the quality of life (Tang et al., 2012). Anterior-only approaches, posterior-only approaches, or 360° and 540° reconstructions are normal surgical options to correct the deformity (Nottmeier et al., 2009), among which, the anterior approach is of importance in correcting the kyphosis (Ebot et al., 2020). However, Park et al. (2019) conducted a retrospective cohort study on discectomy and found that multi-level fusion was significantly associated with the increased risk of screw failure (*p* < 0.01). Screw loosening and plate migration in the anterior cervical discectomy fusion (ACDF) is not rare (Ning et al., 2008; Guo et al., 2021), even though hardware failure attributed to ACDF was demonstrated only 0.1%–0.9% in the United States (Epstein, 2019). Many clinical case reports revealed that screw loosening and anterior cervical plate migration happened in ACDF (Azadarmaki and Soliman, 2014; Nathani et al., 2015; Wójtowicz et al., 2015; Fryer et al., 2019; Ansari et al., 2020). Screw loosening in ACDF is one of the most dangerous complications in cervical anterior plating fixation, which may lead to severe consequences such as esophageal perforation and bone nonunion (Ning et al., 2008; Hershman et al., 2017; Fryer et al., 2019). Therefore, prompt recognition and effective foreseeing of the potential pharyngoesophageal perforation would be pretty helpful in reducing the mortality and morbidity.

Previous clinical research and biomechanical research studies help make decisions but not to improve surgical approaches. Furthermore, few studies stated a detailed FE modeling strategy from a numerical computation and biomechanical perspective for reference and few proposed a practical criterion to evaluate the biomechanical outcomes of the ACDF. Researchers reported an in-silico analysis of the cervical-related surgery and concluded various treatment suggestions based on the reduced Von Mises stress of joint facets or endplates, restricted ROMs, etc. (Ng and Teo, 2001; Li et al., 2017; Ouyang et al., 2019, 2020; Zhou et al., 2022), while limited investigations demonstrated the effective in-silico method to predict the surgical outcomes of deformity correction. Moreover, in most existing in-silico studies, the modeling method and the computational consideration have not been stated clearly, which may leave confusion for replication.

In the present investigation, a finite element analysis (FEA) and the principal–strain based criterion were proposed to capture the biomechanical response and damage evaluation of ACDF, as well as computational, biomechanical, and anatomical explanations of the modeling strategy, providing a numerical solution for surgeons to improve the surgical approach and determine instrument configuration.

## 2 Finite element model

### 2.1 Modeling strategy and material properties

**FIGURE 1:**
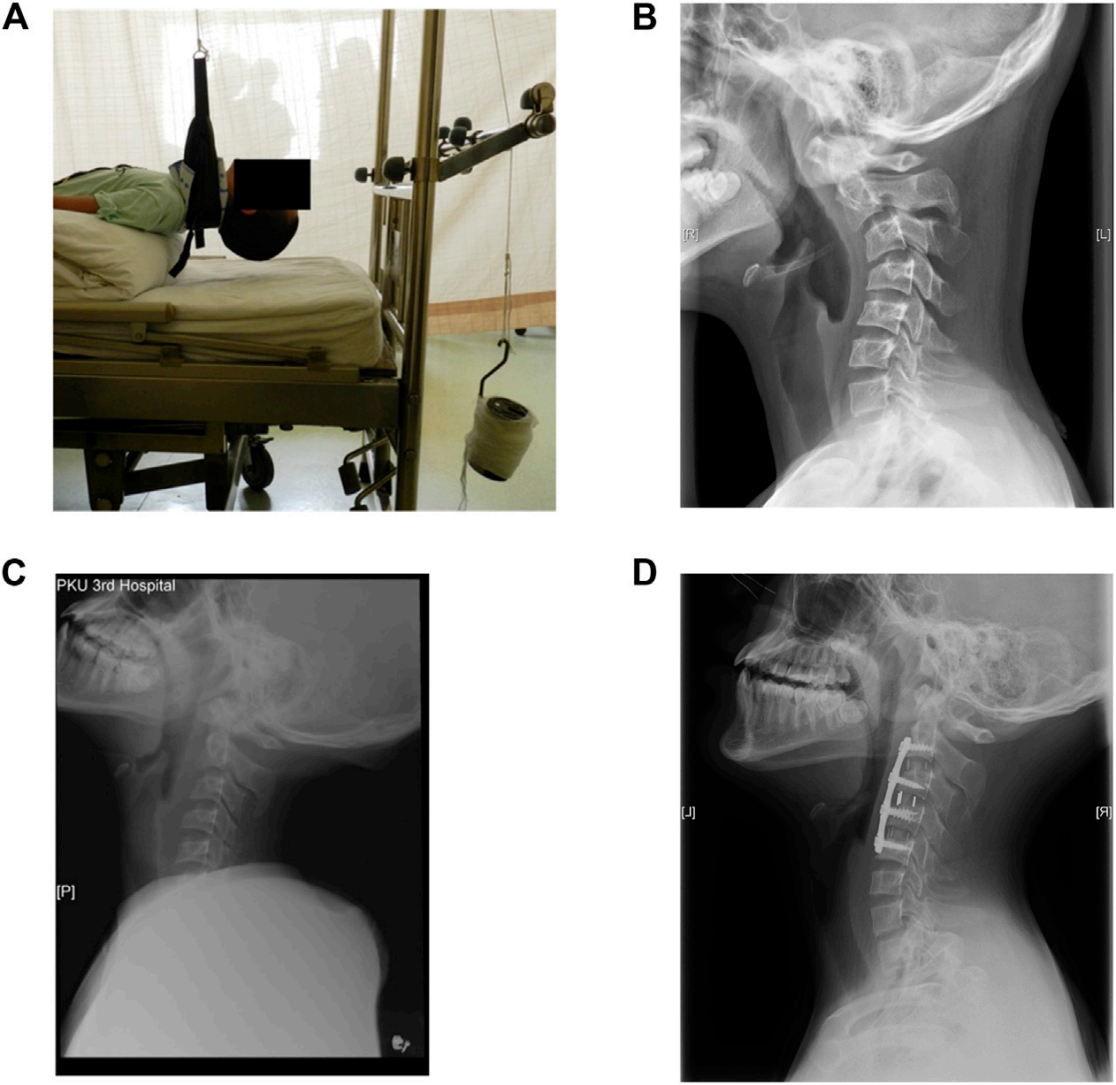
**(A)** Photo of the preoperative moment-balanced traction, **(B)** lateral view of preoperative cervical X-ray, C2–C5 Cobb angle was −53.2°, **(C)** X-ray after the moment-balanced traction, **(D)** lateral view of cervical spine at three-month follow-up, Cobb angle of C2–C5 was −3.3°.

**FIGURE 2:**
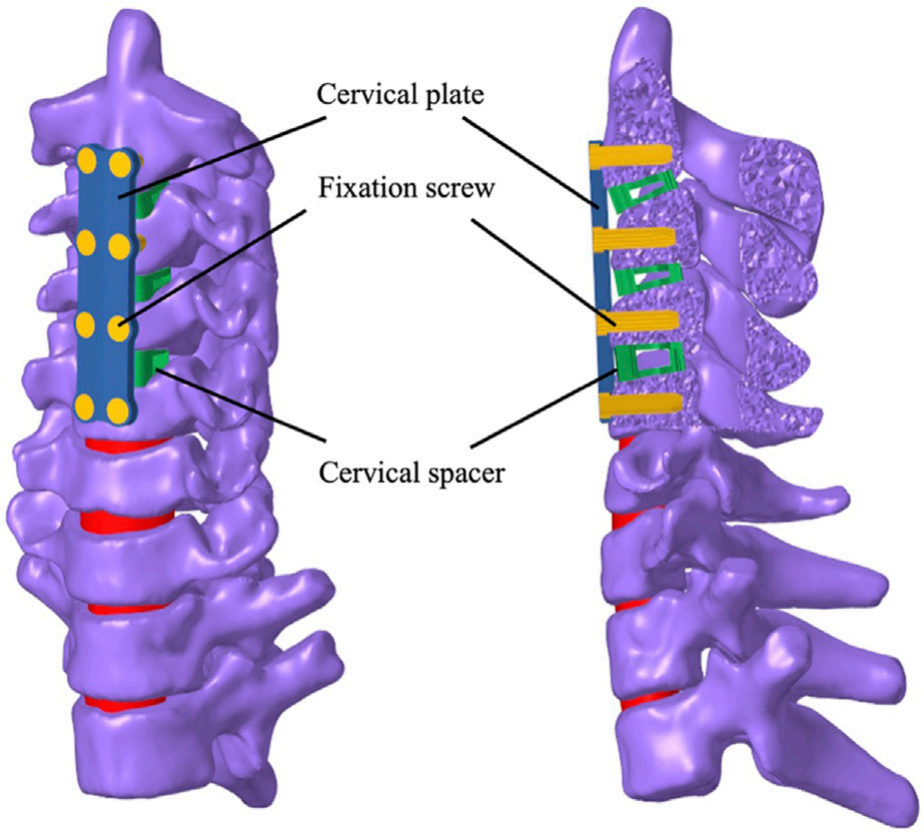
Schematics of the instrument configurations used in the ACDF surgery.

In the present investigation, the computed tomography (CT) data of a 13-year-old patient suffering from cervical kyphosis were obtained, as well as the configuration of the cervical instruments used in the ACDF surgery. Preoperative moment-balanced traction was performed to stretch and relax the anterior muscles for 4 days; photo and X-ray were taken before and after the moment-balanced traction (Figures 1A–C). Three cervical spacers of two heights, a four-level ventral plate, and eight variable-angle screws were implanted to the C2–C5 vertebra bodies to correct the kyphosis to a Cobb angle of 0°, details are listed in Table 1. The cervical ventral plate is manufactured by Depuy Synthes (Raynham, MA, United States) and the cervical ventral plate and fixation screws are from Medtronic (Memphis, TN, United States), as shown in Figure 2. Anterior longitudinal ligaments from C2 to C5 were resected due to the placement of the implant. A three-month follow-up demonstrated that the postoperative Cobb angle of C2–C5 was maintained at −3.3° (Figure 1D) compared with the preoperative C2–C5 Cobb angle of −53.2° (Figure 1B).

**TABLE 1:** Implant configuration in the ACDF surgery. Intervertebral disc (IVD).

	Dimension (mm)	Material	Applied location	Product catalog
Lordosis cervical spacer	8 mm (Height, standard, lordosis)	PEEK	C2-C3 IVD, C3-C4 IVD	Depuy Synthesis Cervios
Lordosis cervical spacer	7 mm (Height, standard, lordosis)	PEEK	C4-C5 IVD	Depuy Synthesis Cervios
Fixed-angle screws	4.0 × 15.0 (D × L)	Titanium	C2, C3, C4 and C5	Atlantis Vision Elite
Anterior cervical plate	55 mm (total length)	Titanium	C2, C3, C4 and C5	Atlantis Vision Elite

During the reconstruction of the cervical–thoracic spine (from C2 to T2), preoperative CT scans were put into a 3D slicer (http://www.slicer.org) to perform the geometrical reconstruction of each vertebra, in which the trabecular bone was identified via a seed-growing method. The cortical layer will be built when mapping the mesh. Then, the cervical instruments were put into the exact positions as per postoperative CT scans, followed by the positional adjustment of the reconstructed vertebrae. Boolean operations allow the model to consider the grinding manipulation on the endplates of vertebra bodies. Thus, the postoperative spine-implant system was acquired. Geometries were then transferred to Hypermesh 2020 (Altair Technologies, Inc., CA, United States) and Jung and Bhutta, 2022 (Abaqus, Inc., Providence, RI, United States) to do the mesh work and perform the finite element (FE) analysis.

**FIGURE 3:**
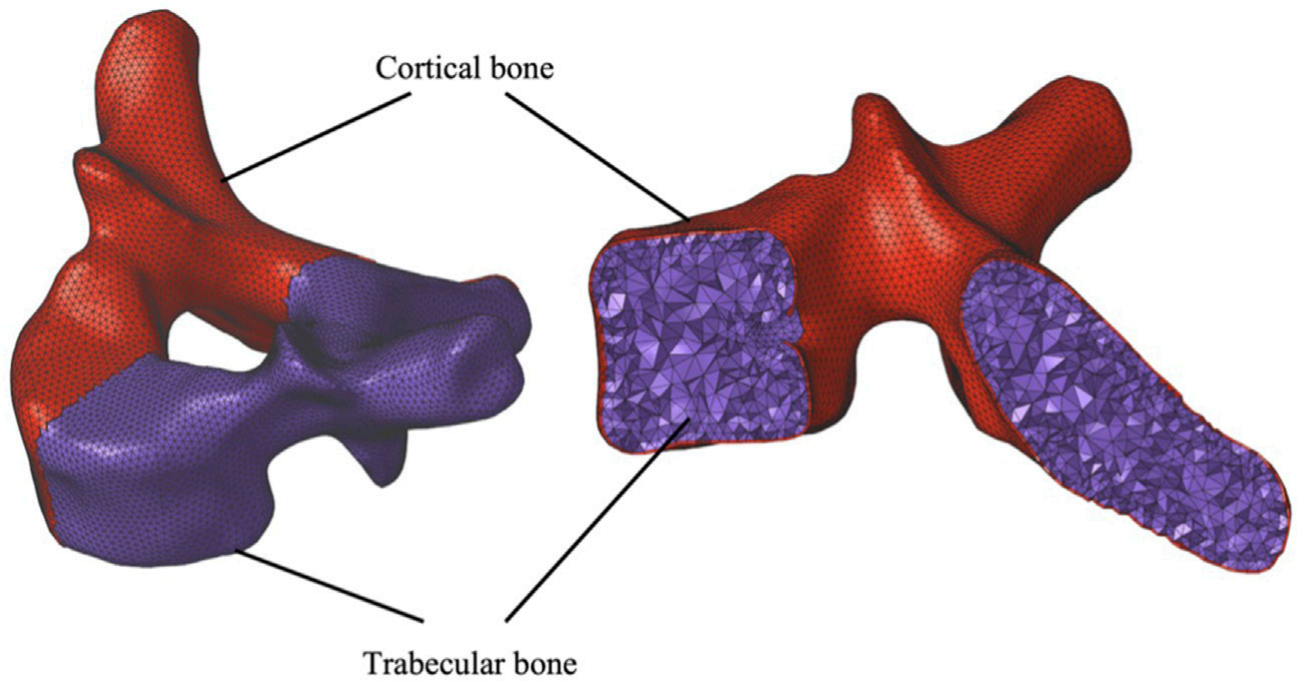
Segmentation of the trabecular bone and cortical bone in the vertebra.

**FIGURE 4:**
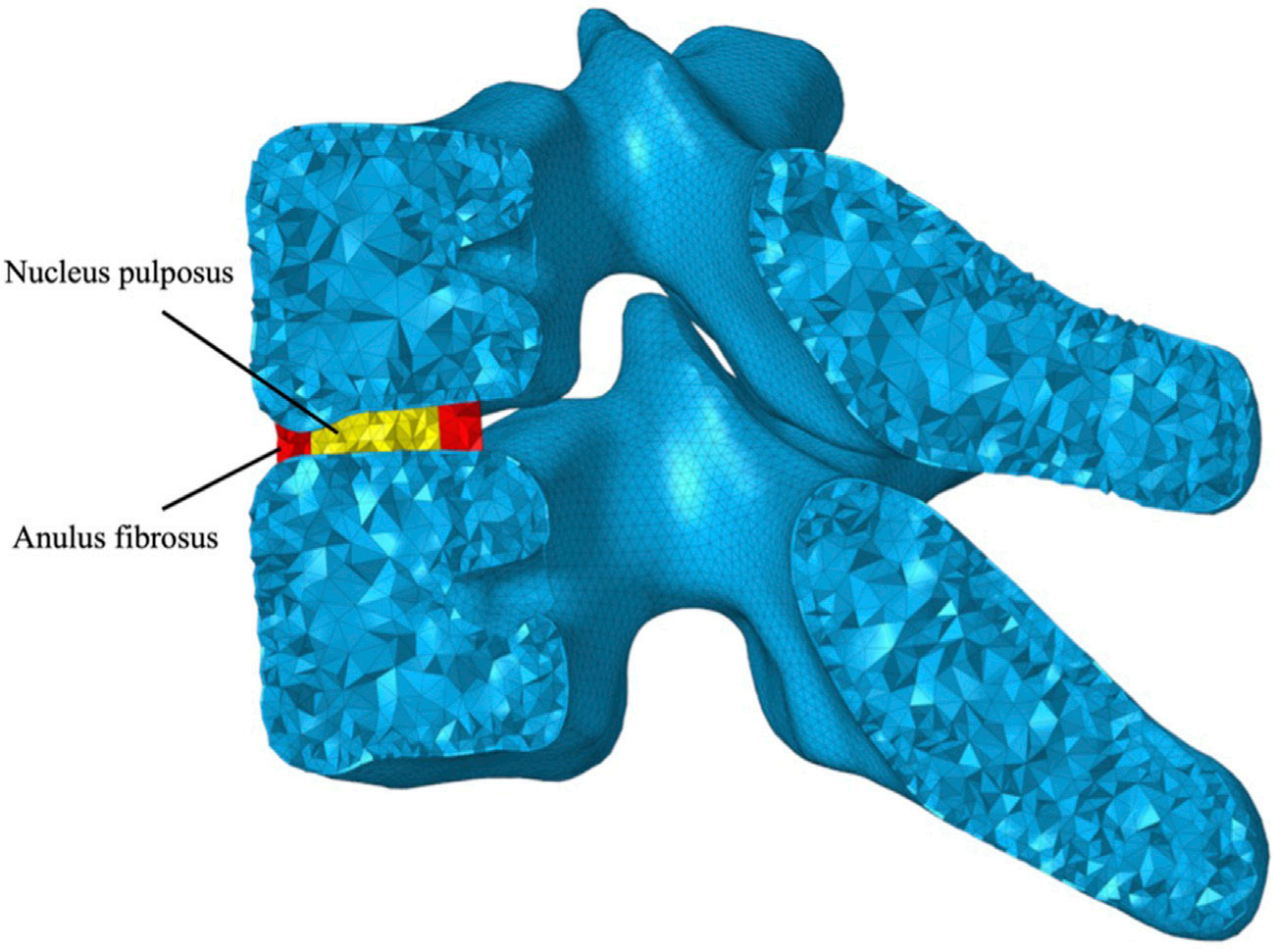
Graphical representation of the intervertebral disc (IVD) consisting of nucleus pulposus (NP) and anulus fibrosus (AF).

Following the representation of the trabecular bone volume, a layer of triangular prism elements was offset from the outer surface, corresponding to the cortex of each vertebra, 0.28 mm thickness for the cervical vertebrae and 0.24 mm for the thoracic vertebrae (Ritzel et al., 1997) (Figure 3). Intervertebral discs (IVDs) were reshaped based on the superior and inferior surfaces of the adjacent vertebral bodies, in which the anatomic structure including the inner nucleus pulposus and outer anulus were reconstructed, shown in Figure 4. Nucleus pulposus (NP) covers a total of 25 %–50 % of the area of the superior and inferior surfaces and accounts for 40 %–50 % of the intervertebral disc volume (Farfan et al., 1970; Pooni et al., 1986; Oegema, 1993; Iatridis et al., 1996; Nachemson, 2014; Perey, 2014). Typical architectures of IVDs were reconstructed for simplification, although IVDs in the cervical spine lack a concentric anulus fibrosus around their entire perimeter (Mercer and Bogduk, 1999). Superior and inferior surfaces on NP and anulus fibrosus (AF) were tied onto the inferior surface of the superior vertebra and the superior surface of the inferior vertebra, respectively, forcing all translational and rotational degrees of freedom (DOFs) to be the same. Linear elastic mechanical properties of NP and AF were defined to level down the non-linearity of the FE model (Table 2) (Ng and Teo, 2001).

**TABLE 2:** Mechanical properties of the bones, IVD components, and implants in the FE model.

		BMD (mg/cc)	Young’s modulus (MPa)	Poisson’s ratio
C2 trabecular	Central vertebra body	200.4	403.10	0.30
	Exterior vertebra body	135.2	182.75	
C3 trabecular	Central vertebra body	242.1	589.43	
	Exterior vertebra body	99.7	99.08	
C4 trabecular	Central vertebra body	319.2	1,027.46	
	Exterior vertebra body	185.9	346.62	
C5 trabecular	Central vertebra body	301.3	914.93	
	Exterior vertebra body	251.5	636.33	
C6 trabecular	Central vertebra body	262.6	694.04	
	Exterior vertebra body	141.8	201.13	
C7 trabecular	Central vertebra body	229.1	527.53	
	Exterior vertebra body	124.7	155.34	
T1 trabecular	Central vertebra body	165.7	275.07	
	Exterior vertebra body	55.1	30.08	
T2 trabecular	Central vertebra body	187.4	352.26	
	Exterior vertebra body	140.4	197.16	
Cortical bone			1,000	0.30
Nucleus pulposus			1.0	0.49
Anulus fibrosus			3.4	0.40
PEEK			4,000	0.35
Titanium			110,000	0.30

The linear elastic material model was defined to describe the biomechanical behavior of the trabecular bone and cortical bone, which was evaluated through a phantom-less bone mineral density (BMD) measurement (Liu et al., 2022); then, BMD was converted to Young’s modulus as per Eq. 1 (Keyak et al., 1997), allowing a patient-specific and BMD-dependent analysis. The mechanical properties of the cortical bone were obtained from existing literature studies, since the cortex layer in CT cannot be computed to obtain the BMD given the thickness was about 0.3 mm. The curvature of the cervical spine and the force transition path shaped a non-uniform BMD distribution—BMD of the central vertebra body and exterior vertebra body are significantly different (Anderst et al., 2011; Feng et al., 2021). Therefore, BMD was measured separately in the central vertebra body and exterior vertebra body.

$$\left\{ \begin{array}{l} E=0.001\left(BMD=0.00\right)\\ E=33900\times {\left(\frac{BMD}{1000}\right)}^{2.2}\left(0.00<BMD<0.27mg/cc\right)\\ E=5407\times BMD+469\left(0.27mg/cc\leq BMD\leq 0.60mg/cc\right)\\ E=10200\times {\left(\frac{BMD}{1000}\right)}^{2.01}\left(0.60mg/cc\leq BMD\right) \end{array}\right.$$ (1)

Modified quadratic tetrahedral elements (C3D10M) and quadratic triangular prism elements (C3D15) were used to map the vertebral bodies. Geometry-editing tools, including edge toggling and edge combining, were employed to clean the geometry and acquire an optimum mesh, though mild topographical deviation might be introduced. A hexahedron mesh mixed with prism mesh was created for screws, meshes of cervical plates, and spacers were mapped through delicate geometry partitions and symmetrical mesh generation. To minimize the numerical deviation brought by the mesh density, element aspect ratio, volume skewness, and tetrahedral collapse indices were inspected following the mesh mapping; Table 3 lists mesh quality indices for every vertebra. Convergence studies were conducted for every vertebra and IVD individually, and the final mesh size for vertebrae was set to 0.8–1.2 mm, and 0.8–1.5 mm for surgical instruments. Node equivalence between the bones and instruments were performed to avoid errors affected by the interpolation of node–node variables in node–node constraints theoretically.

**TABLE 3:** Mesh quality inspection criterion and the percentage of fair-quality element.

	Jacobian less than 0.7	Volume skew higher than 0.95	Tetra collapse higher less 0.1
C2	0.00% (min 0.65)	0.00% (max 0.89)	0.00% (min 0.12)
C3	0.00% (min 0.71)	0.00% (max 0.89)	0.00% (min 0.10)
C4	0.00% (min 0.65)	0.00% (max 0.90)	0.00% (min 0.10)
C5	0.00% (min 0.67)	0.00% (max 0.89)	0.00% (min 0.10)
C6	0.10% (min 0.61)	0.00% (max 0.89)	0.00% (min 0.10)
C7	0.00% (min 0.69)	0.00% (max 0.89)	0.00% (min 0.10)
T1	0.00% (min 0.55)	0.00% (max 0.89)	0.00% (min 0.10)
T2	0.00% (min 0.65)	0.00% (max 0.89)	0.00% (min 0.11)

**FIGURE 5:**
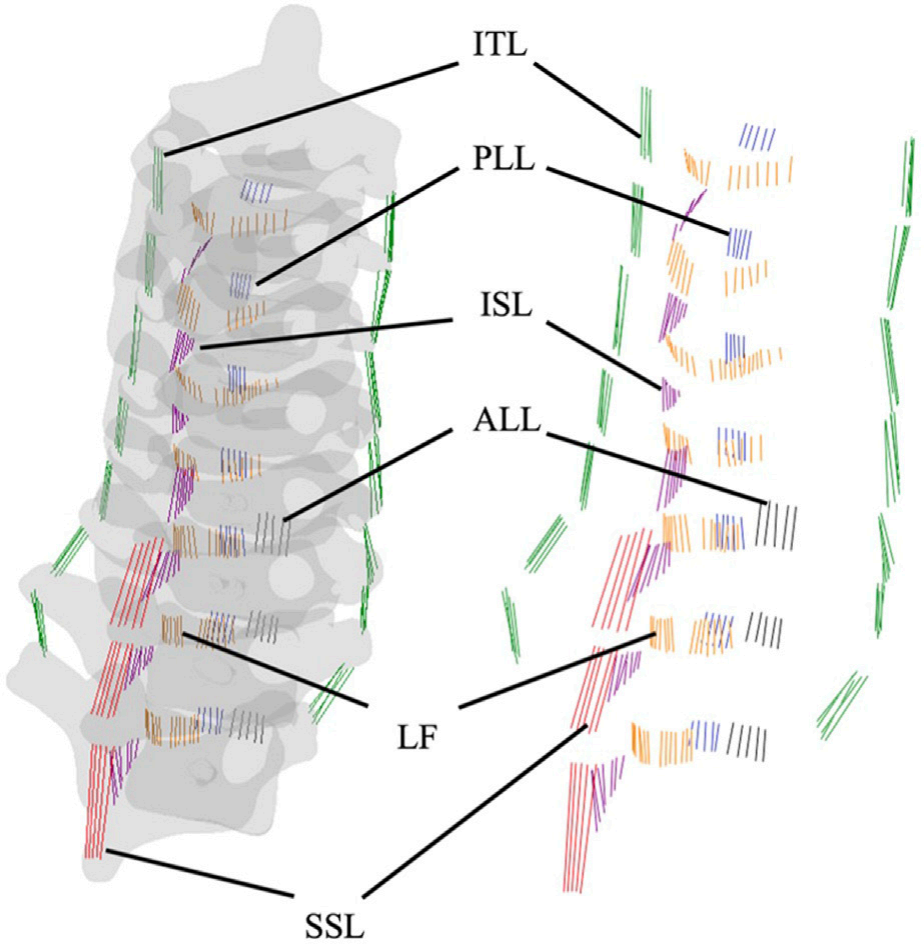
Anatomical schematics of ligaments and truss elements in the FEA model.

Several truss elements were incorporated to represent major connective tissues. The posterior longitudinal ligament (PLL), anterior longitudinal ligament (ALL), interspinal ligament (ISL), supraspinal ligament (SSL), intertransverse ligament (ITL), and ligamentum flavum (LF) were built to simulate the force transition. Partial SSL was taken into consideration, as the remaining SSL were attached on ISL; miss-representation would import an extra system error to the numerical analysis. Only one truss element (T3D2) was mapped for every unit in each ligament, of which only the tension load would be effective and no moment being transited. Figure 5 illustrates both the attachment of ligaments and the graphical representation in the FE model, the transverse area of each ligament and mechanical properties are listed in Table 4 (Shirazi-Adl et al., 1984; Lu et al., 1996).

**TABLE 4:** Cross-section area of every ligament and mechanical property.

	Young’s modulus (MPa)	Poisson’s ratio	Cross-section area (mm^2^)
Anterior longitudinal ligament (ALL)	20	0.3	38
Posterior longitudinal ligament (PLL)	70		20
Interspinal ligament (ISL)	28		35.5
Supraspinal ligament (SSL)	28		35.5
Intertransverse ligament (ITL)	50		10
Ligamentum flavum (LF)	50		60

Joint facets within C2–C5 were difficult to identify due to the deformity of exterior vertebra bodies; cervical spacers and a cervical plate were placed and would carry most loads; besides, the reconstructed gap between the cervical articular processes was uneven in every joint. Therefore, the cartilage layers were excluded in the proposed FE model. Surface-to-surface, small-sliding contacts were set at the contacting area with a 0.07 friction coefficient and no penetration behavior was allowed, covering from the C5–C6 segment to T1–T2 segment. The finite-sliding contacting method was excluded considering the huge computational burden.

The head weight (HW) of the patient was 7.83% estimated by the averaging head weight percentage of human body weight (Ramachandran et al., 2016). Anatomically, HW will be transited through the occipital condyle to massa lateralis of C1, then loaded onto C2. Thus, a 30.7 N gravity load was applied onto both sides of the cartilage facets next to the odontoid process in C2. The present model neglects all muscles surrounding the cervical and cervical-thoracic segments, torque under different motions were estimated according to an isometric-strength experiment to simulate the daily cervical motion postoperatively (Kauther et al., 2012). The motion of the entire upper body was excluded (flexion, extension, and lateral flexion) in the present investigation, only the motion in cervical columns was considered, since the acceleration of the entire upper body cannot be estimated. Applied torque values are listed in Table 5, which were calculated by multiplying HW by the isometric cervical strength. The torque was applied on the spinous processus, and kinematic-based coupling was utilized to minimize the undesired deformation caused by the applied torque in C2.

**TABLE 5:** Torque applied under different loading conditions.

	Isometric cervical strength (Kauther et al., 2012) (Nmm/kg)	Applied torque (Nmm)
Flexion	418	1,308
Extension	683	2,138
Lateral flexion	542	1,696
Rotation	208	651

The aforementioned FE configuration led to a computational time of less than 150 mins with a workstation of an i7-10700K (3.79 GHz) processor with 78 GB RAM in use.

### 2.2 Failure criterion

Principal strain was utilized to determine whether the bone was unable to bear the shear force or compression force caused by the fixation screws. If the tension principal strain (maximum component) in one bone element reaches 1.5 %, or the compression principal strain (minimum component) meets −2.0 % (Soyka et al., 2016), then that element is regarded as broken. The strain limit used here was taken from the study toward lumbar vertebrae, since the BMD in the present study is much higher than in the lumbar segments in Rene’s research. The damage index of every bone element is defined as the maximum of the ratio between the element principal strains (ε_1,i_ or ε_3,i_) and the corresponding principal strain threshold (ε_t,t_ or ε_c,t_), i.e.,

$$D_{i}=\max\left(\frac{\varepsilon_{1,i}}{\varepsilon_{t,t}},\frac{\varepsilon_{3,i}}{\varepsilon_{c,t}}\right)$$ (2)

### 2.3 Validation

The proposed FE model was validated against the retrospective medical data of the patient directly and indirectly. The four-month postoperative CT images showed that the purchase of the fixation screws was good; no screw loosening or spacer subsidence was observed. After seventeen months, the surgery outcomes were good till the numerical investigation was performed. Inspecting the damage indices in the central vertebra body of each vertebra, bone elements surrounding the screw trajectories, and cage-contacting facet were all below the threshold of one under all four loading conditions, maintaining good fixation and forming a good fusion.

As small-sliding contacts were utilized to include joint slipping, contact slipping was examined thoroughly to determine the reasonability of small-sliding. The largest slip motion in slave nodes was below 0.8 mm, only three slave nodes (about 0.00%) displaced larger than the length of the element and reached 1.2 mm. No contact chattering and large node adjustments were found in the analysis according to the analysis log.

Computed damage indices showed good consistency with the surgical outcomes, calling for the acceptance of the proposed FE model’s outputs and modeling strategy. Valuable insight to the influence of the cervical implant is discussed, as well as the biomechanical response toward various loadings in the following sections.

## 3 Results

### 3.1 Postoperative cervical stability

**FIGURE 6:**
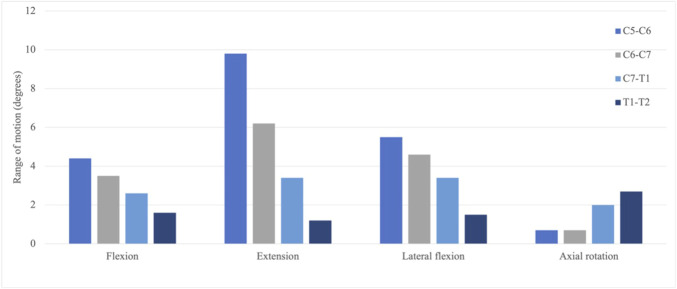
ROM of the intact joints under different loading scenarios.

Most studies of the lower cervical spine have addressed flexion and extension movements, for these are the cardinal movements exhibited by these segments (Anatomy, Head and Neck, Neck Movements—StatPearls—NCBI Bookshelf). As shown in Figure 6, the range of motions (ROMs) of the non-surgical segments in the flexion, extension, and lateral flexion show a declining trend, especially in the extension motion (around 40% decrease), while an increasing trend is seen in the axial rotation.

ROMs of the C5–C6 segment under flexion, extension, lateral flexion, and axial rotation were 4.4°, 9.8°, 5.5°, and 0.7°, respectively; ROMs of the C6–C7 segment, 3.5°, 6.2°, 4.6°, and 0.7°, respectively; ROMs of the C7–T1 segment, 2.6°, 3.4°, 3.4°, and 2.0°, respectively; and ROMS of the T1–T2 segment, 1.6°, 1.2°, 1.5° and 2.7°, respectively.

**FIGURE 7:**
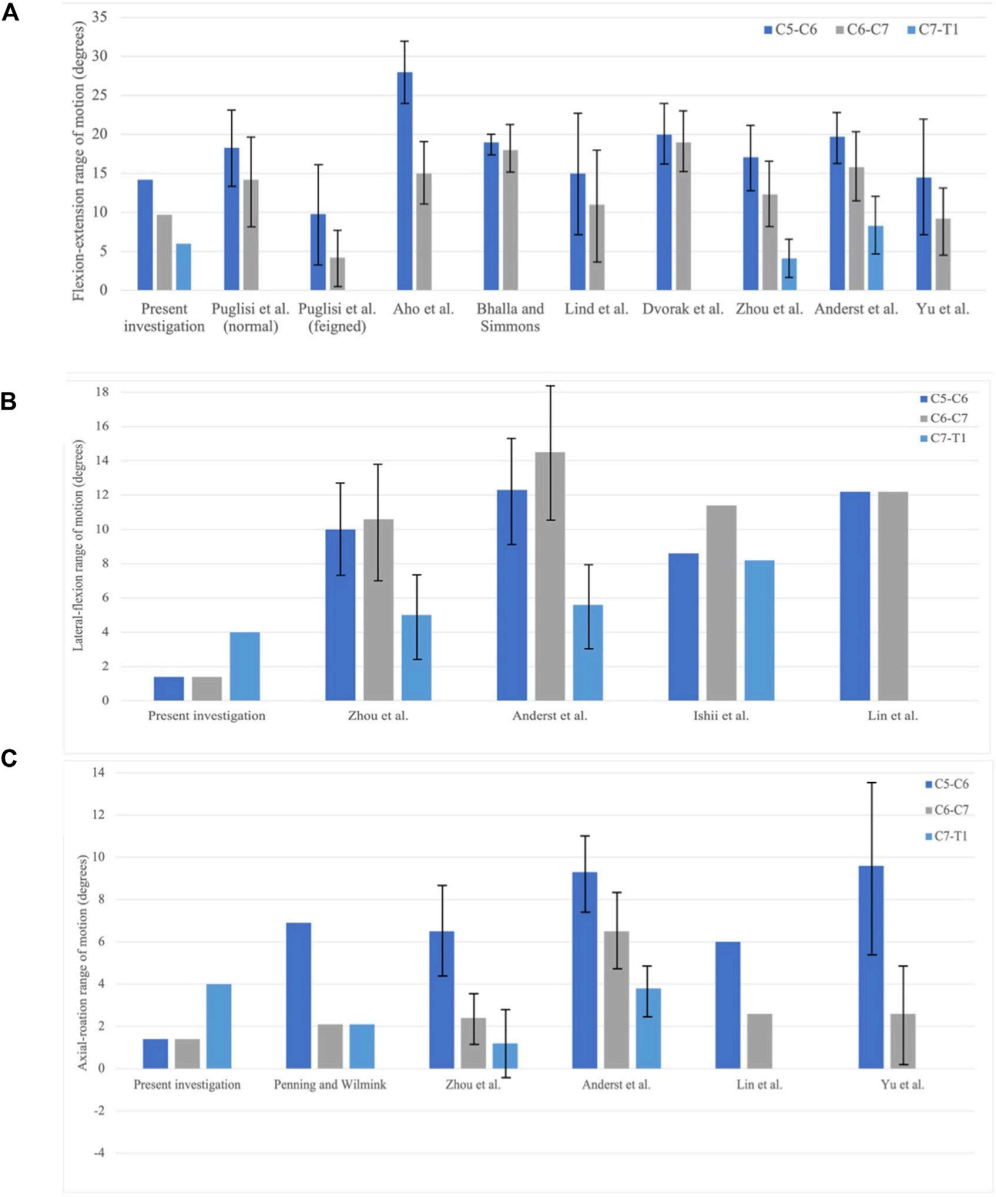
Comparison of the range of motion (ROM) in between the present investigation and experiment results. C5–C6, C6–C7, and C7–T1 joints are collected. **(A)** Flexion–extension; **(B)** lateral–flexion; and **(C)** axial rotation (Bogduk and Mercer, 2000; Puglisi et al., 2007).

**FIGURE 8:**
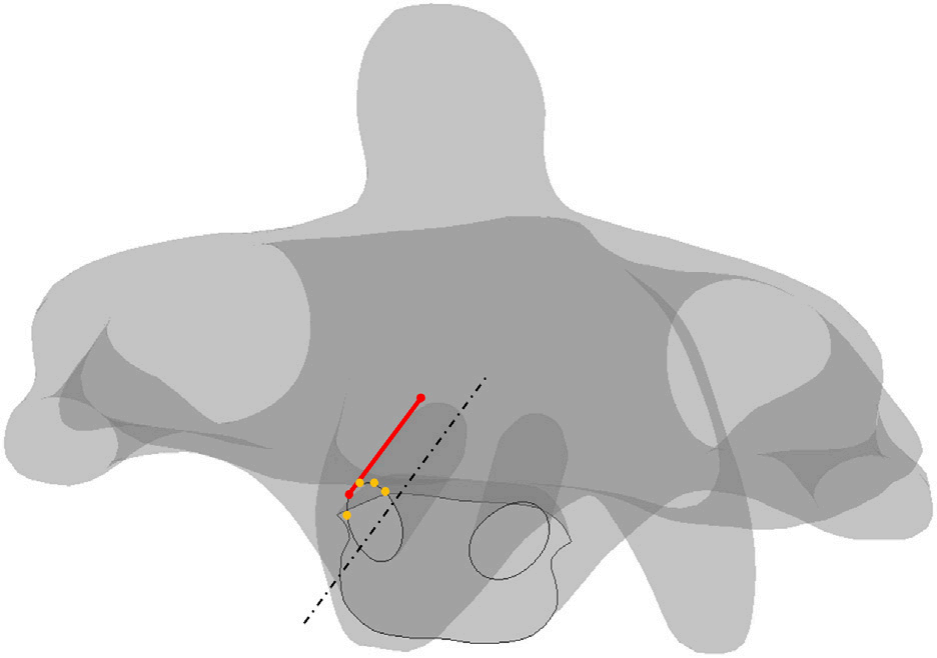
Schematics of screw plowing in the cervical screw fixation. Lines in dash form are for the reference, points in orange are on the trajectory, the line in red represents the shortest length from the anterior section to the posterior section.

Compared with the experimental results of asymptomatic subjects, computed ROMs of the C5–C6 segment and the C6–C7 segment in flexion–extension are below the mean ROM obtained from the literature, despite that Yu et al. (2019) recorded a 9.2 ± 4.3° in the C6–C7 joint (Figure 7A). The ROM in the flexion–extension of the C7–T1 segment in the present study falls into the mid of Zhou et al. (2020) and Anderst et al. (2015), 6.0°, 4.1°, and 8.3°, respectively. The decreasing percentage of the ROM in the present study is 32% and 38%, while Zhou captured 28% and 67% decrease percentages, and Anderst saw 20% and 47% decrease percentages. Figure 8 demonstrates a restricted lateral–flexion motion after the ACDF. The computed ROMs acquired the same lateral flexion at 1.4°, while the experimental results exhibited nearly the same ROMs for C5–C6 and C6–C7 segments; the C5–C6 segment flexed slightly smaller than the C6–C7 segment in experiments (Figure 7B). When the neck was subjected to an axial-rotation load, the computed ROMs of the C5–C6 segment moves little in contrast to the largest rotation measured in experiments, as shown in Figure 7C.

### 3.2 Mechanical response of bone

The absolute-maximum principal strain and deformation around the screw trajectories in four types of motion are exhibited below, where C2 always has the largest deformation among the four motions. Notably, the largest deformation in every trajectory concentrated to the segment on the anterior section where the short length of the point to the screw tip (Figure 8) screw-plowing is happening in cervical spine fixation.

#### 3.2.1 C2

**FIGURE 9:**
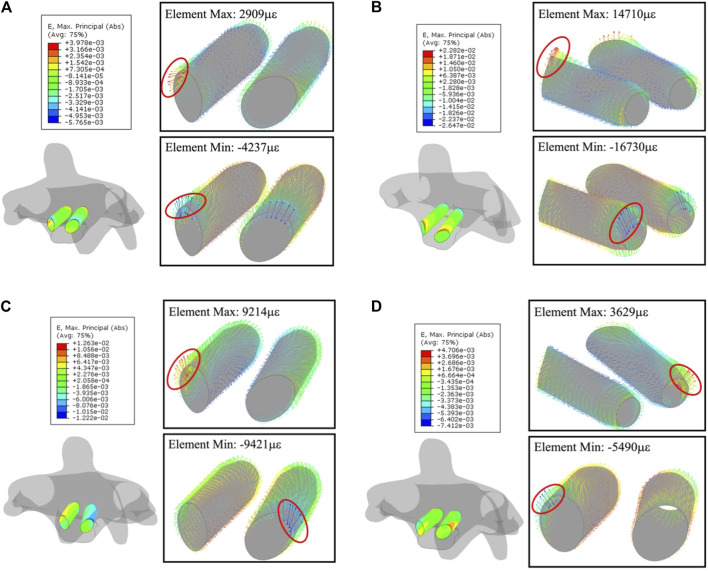
Principal-strain and deforming direction surrounding the trajectories in C2. **(A)** Flexion, **(B)** extension, **(C)** lateral flexion, and **(D)** axial rotation. Left figure in each motion is the strain on nodes, figures within the box display the strain in each element.

The largest compression and tension deformation were observed in the upper half of the anterior section, where the largest deformations of 1.4710 με (1.47%) and −1.6730 με (−1.67%) were recorded under the extension. Figure 9 gives the overall strain distribution in trajectories under every motion, including the absolute-maximum principal strain on nodes (figures outside box) and the principal strain in every bone element (figures inside box).

#### 3.2.2 C3 and C4

Deformation on the trajectories in C3 and C4 are at the same level, slightly lower than in C5. In C3, maximum and minimum values in each element for flexion, extension, lateral flexion, and axial rotation are (689.2, −996 με), (1681, −4,114 με), (1,742, −1,889 με), and (1,923, −1,325 με), respectively; in C4, (675.6, −755.9 με), (1,421, −2,451 με), (1,211, −1,474 με), and (1,586, −1,054 με), respectively.

#### 3.2.3 C5

**FIGURE 10:**
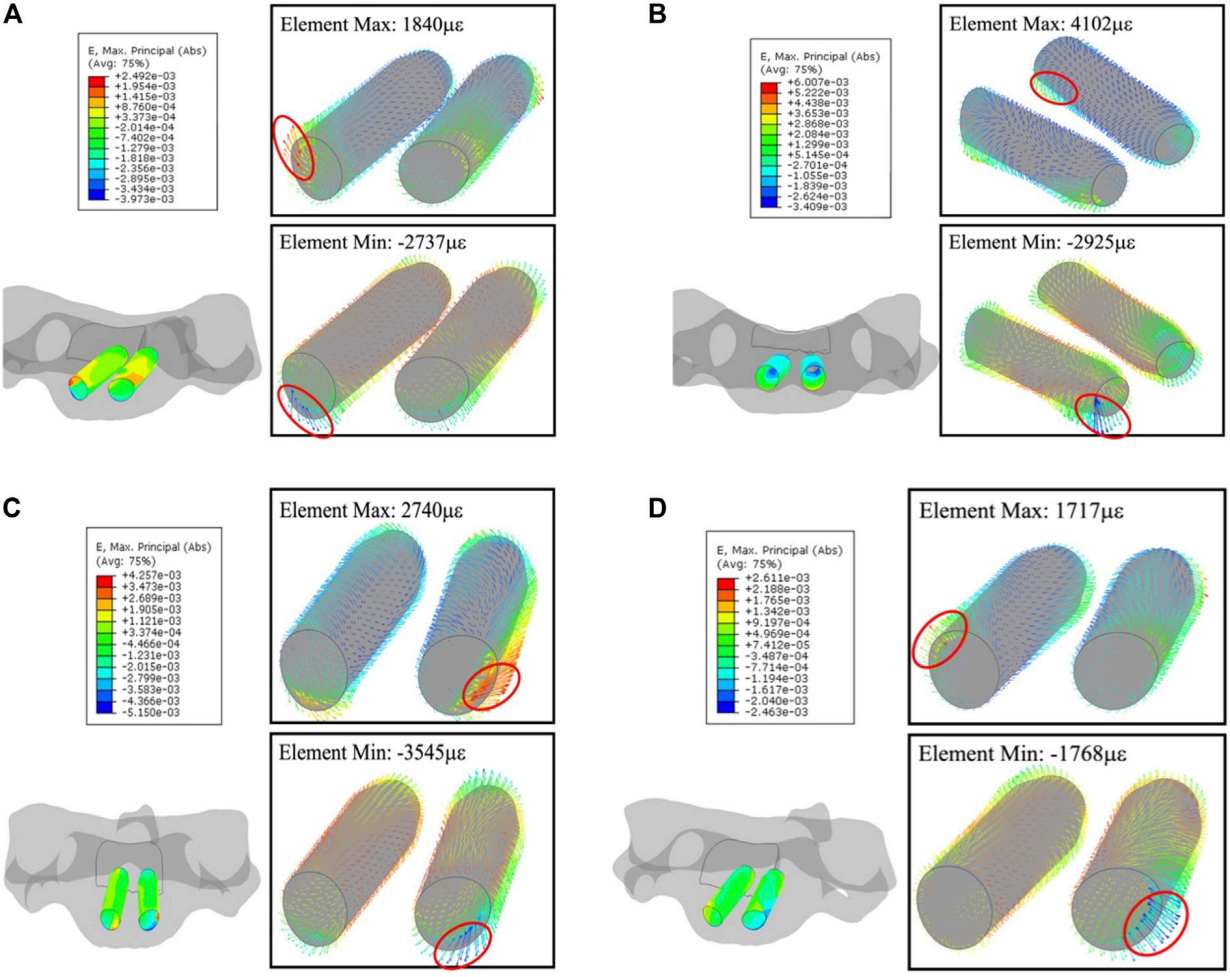
Principal-strain and deforming direction surrounding the trajectories in C5. **(A)** Flexion, **(B)** extension, **(C)** lateral flexion, and **(D)** axial rotation. The left figure in each motion is the strain on the nodes, figures within the box display the strain in each element.

Like C2, the strain concentrates to the segment on the anterior section of the trajectory due to the short length to screw tip planar; (1,840, −2,737 με) in flexion, (4,102, −2,925 με) in extension, (2,740, −3,545 με) in lateral flexion, and (1,717, −1,768 με) in axial rotation, respectively. Figure 10 shows such a strain pattern.

## 4 Discussion

### 4.1 Modeling method

Connective soft tissues in the human body are variables that depend on biological parameters, it is impossible to describe them accurately for individuals in clinical–biomechanical research. In the present FE investigation, the boundary conditions including the loads simulating the physical flexors and extensors were created based on existing well-designed biomechanical experiments and anatomical inspections. When applying the moment that forces the neck to do motion, the driving muscles are taken seriously, even though muscles are replaced with a moment. Anatomically speaking, the three main groups of cervical muscles surrounding the vertebral columns control the neck motion, which are the cervical flexors, the cervical extensors, and the sub-occipital muscles. The sub-occipital muscles dominate the motion in the cranio-cervical joint, thus they are skipped in the discussion section.

The cervical flexors consist of sternocleidomastoid (SCM) and anterior scalenes (AS); the SCM travels obliquely across the side of the neck and inserts at the skull (Robinson and Anderson, 2005; Gray and Grimsby, 2012) and the AS origins from the cervical vertebrae C3–C6. The AS functions as a contracting motor that allows the neck to bend forward, flexing laterally and rotating; however, to the best of the author’s knowledge, motion contribution of every vertebra (C3–C6) is unclear and makes the model unable to replicate the contracting effect of the AS. The deep flexor muscles mainly refer to the longus colli and longus capitus. The longus colli is regarded as a weak flexor, the longus capitus controls the cranio-cervical flexion and supports the cervical lordosis anteriorly (Falla et al., 2004; Jull et al., 2008). Consequently, the deep cervical flexors are excluded in the FE model.

The extensor group is described as having four layers (Schomacher and Falla, 2013): first layer, levator scapulae and upper trapezius; second layer, splenius capitus and cervicis; third layer, semispinalis capitus; and fourth layer, semispinalis cervicis and multifidus. The extensor group controls the flexion, extension, lateral flexion, and rotation of the neck (Cleland et al., 2015).

Hence, the moment that forces the neck to move was applied onto C2 and was coupled to a large sum of mesh nodes around the spinous process, distributing the moment to partial C2. Such a load-applying method is inherently flawed as only C2 was subjected to the load yet no more direct load was applied onto the lower cervical vertebrae. Various loading conditions are applied to perform the FE analysis in the previous research. Ouyang et al. (2020) applied a physiological compression of about 7.5 kg and 1,000 Nm moment on the superior endplate of the C3 vertebra in the intact model, then applied the movement angle acquired in the intact model to the ACDF model (Ouyang et al., 2020). The 1,000 Nm moment in their research kept the same magnitude as the load applied in the Panjabi et al. (2001), allowing the comparison of ROMs between the *in silico* model and *in vitro* model. Zhang et al. (2006) forced the skull to move along various anatomical planes with a 1,000 Nm moment, while Li and Lewis (Li and Lewis, 2010) applied 1,000 Nm onto the superior surface of the C1 vertebra. In reported biomechanical experiments, Panjabi et al. (1991) loaded a 1,000 Nm moment to cervical spine segments, in which, the 1,000 Nm was able to cause physiological motions without any damage; Wheeldon et al. (2006) tested the cervical spine segments with 330, 500, 1,000, and 1,500 Nm under the flexion–extension motion. Various loading conditions resulted in motions in the direction of loading under within the elastic range, exposing the primary kinematics and biomechanical response (Moroney et al., 1988; Oda et al., 1991; Panjabi et al., 1991, 1994; Crawford et al., 1998; Wilke et al., 1998; Winkelstein and Myers, 2000, 2002). The moment calculated based on the isometric strength is varied under different motions, similar to the measurement in the Rezasoltani’s experiment, the moment applied here is smaller than in Rezasoltani’s measurement though (Rezasoltani et al., 2008). The reduced moment covered the daily activities of the neck and produced conservative FE outcomes. Furthermore, the fixation of T1 in the present FE model was slightly offset from the rotation axis defined in the isometric-strength measurement, minimizing the influence of the location of thoracic support (Rezasoltani et al., 2008). The proposed loading conditions incorporated physical information into the FE model and represented the capacity of muscles that maintain a constant length.

### 4.2 Strain-based failure criterion

As far as authors’ knowledge reached, few studies implement the principal-strain method onto the evaluation of the clinical outcomes of deformity correction. The principal strain properly pictures the structural response of the corresponding complex loads and demonstrates the direction of the deformation. Strain, instead of stress, was utilized to define the damage extent of the bone as researchers demonstrated the strain and damage localization early on in the progress were important to bones’ brittleness and the matrix failure of human compact bones were dependent on the local strain type (Boyce et al., 1998; Zioupos et al., 2008). On the other hand, stress cannot be measured directly; if the stress-based threshold was utilized, it may incorporate error of misestimation of Young’s modulus. Typically, the FE method was based on the displacement of the mesh nodes; then the strain field was obtained *via* derivatives of the displacement field, resulting in a higher accuracy of the strain field compared with stress, especially in non-uniform material.

### 4.3 Range of motions and stability

The proposed FE model captured the ROM declines across vertebra segments, especially rapid drops of 40 %–50 % in the extension motion, showing an agreement with the FE outcomes obtained by Ouyang et al., 2019. The flexion and extension in normal individuals are complex and counter-intuitive, as described by Van Mameren (1988). Generally speaking, flexion begins from the lower cervical spine (C4–C7), in which the C6–C7 segment makes maximum contribution; then the C0–C4 block is involved and finally involves the lower cervical spine (C4–C7). Extension is initiated in the lower cervical spine (C4–C7) as well, the C4–C7 block then moves in the regular order of C4–C5, C5–C6, and C6–C7. However, in the proposed FE model, the ventral plate is fixed in the C2–C5 block, thus interrupting the order of contribution of individual segments, impacting the contribution of vertebra segments in flexion and extension motions.

The decreasing trending of the flexion–extension motion of C5–C6 and C6–C7 were also reported in existing biomechanical experiments (Aho et al., 1955; Lind et al., 1989; Puglisi et al., 2007; Anderst et al., 2015; Yu et al., 2019; Zhou et al., 2020). The ROMs in the present investigation were reduced by 40 %–50 % across vertebra junctions, a similar decreasing extent to the results in the feigned group in Puglisi’s research (Puglisi et al., 2007) (Figure 7A). In their experiment, participants were asked to feign a 50 % restricted neck motion, no extra facility was utilized, and no pre-train was conducted though. Postoperative ROMs in the present investigation suggested that the placement of the ventral plate and intervertebral spacer constrained the ROM of the upper cervical vertebrae under flexion–extension. The ventral plate eliminated the relative motion on the processes; meanwhile, the spacer replaced the intervertebral disk, compromising the oblique between the normal of the intervertebral disk and the long axes of the vertebral bodies. Aforementioned restrictions in surgical junctions reduced the moment arm of HW, thus limiting the induced moment on non-surgical junctions.

The lateral-flexion and axial rotation were also restricted due to the ACDF. Therefore, stability is held to improve the segment fusion process and outcome. The restricting effect on flexion–extension ROM in T1–T2 cannot be determined, since the quantitative kinematics data of the cervical–thoracic segments are scarce.

### 4.4 Bone-implant interaction

In C2–C5, large deformations were found in C2 and C5 that are the superior and inferior fixed vertebrae. Furthermore, the deformation around the screws concentrated to the lateral side on the anterior section of the screw trajectory, where an uneven distribution of the distance from the ventral plate to vertebra body was observed. This gap between the ventral plate and bone will induce a moment, resulting in the repeated compression and extrusion on the anterior section of the screw trajectory. Daily activities of the neck will generate a complex loading history and may cause fatigue damage to the bone surrounding the screw trajectory, then the trajectory will be expanded, and screw loosening might happen after a long time. Researchers also demonstrated the excessive strain between the screw and bone interface and considered it as the primary cause for screw loosening (Schizas et al., 2009; Villa et al., 2014). Aycan and Demir (2020) reviewed the cyclic biomechanical experiments on pedicle screws and concluded that the pull-out performance of the screws generally decreased with the toggling effect, emphasizing the strain concentration in the bone–implant interface leading to the loss of the stabilization of fixation. The gap between plate and bones needs to be taken care in the ACDF surgery, and fixation screws should be fully inserted into vertebra bodies.

There are several limitations in the present FE investigation. The cartilage layer was neglected in the analysis, reducing the non-linearity of the relative motion and influencing the contacts between vertebra segments. This simplification introduced an error on the relative motion between two vertebra bodies. Secondly, the truss element was deployed to simulate the ligaments and mechanical properties of normal ligaments that were acquired from the literature; however, the subject in the FE model was diagnosed with kyphosis, anterior soft tissues might be stronger than normal, healthy people. Further work will include more clinical cases into the investigation and validate the proposed principal-strain based on the criterion against retrospective medical records (both successful deformity corrections and failed ones).

## 5 Conclusion

The present *in silico* study proposed a patient-specific method that can be used to inspect the safety of the fixation construct in the cervical spine and explained the modeling strategy in the perspective of mechanics, anatomy, and numerical computation, offering an explanation on the hardware migration. A principal-strain-based failure criterion was introduced to measure the bone failure in the cervical vertebrae body, meanwhile the pre-condition on how to use the criterion was elaborated. The proposed failure criterion of the bone demonstrated satisfying surgical outcomes and was validated against the retrospective inspection, though more clinical validation was needed. Furthermore, the gap between the ventral plate and bone (unsupported threads of the fixation screw) induced moment on the anterior section of the screw trajectory and might cause fatigue damage. Biomechanically, it is recommended that the ventral plate is bent to a conformed shape with the cervical vertebra to reduce the gap between the ventral plate and bone, and the fixation screws are always fully inserted into vertebrae.

## Data Availability

The datasets presented in this article are not readily available because requests to access the datasets should be directed to wuuuhku@connect.hku.hk.
